# Investigation and Improvement Strategies for Mold Fracture: A Study on the Application of a Pulse Electrodeposition Method for Enhancing Mold Lifespan

**DOI:** 10.3390/ma16237291

**Published:** 2023-11-23

**Authors:** Xionghua Jiang, Fengyu Kong

**Affiliations:** Department of Metal Materials, College of Materials Science and Engineering, Dongguan University of Technology, Dongguan 523000, China; fengyu.k@hotmail.com

**Keywords:** mold, fracture, pulse electrodeposition, NiCo coating

## Abstract

An investigation on the fracture of a mold, comparing it with a normal part using specific techniques, such as EDX, SEM, and AES, is presented in this study. The EDX analysis revealed that the composition of the normal part was consistent with that of low-carbon steel, mainly comprising Fe and C. In contrast, the fractured part exhibited cracks due to nonconforming nonmetallic inclusions and reticular carbides, with fractures resulting from microporosity agglomeration and cleavage fracture. The SEM and AES analyses further presented the causes of mold fracture, highlighting the mechanism by which the dimples on the specimen edge contributed to the fracture. The EDX analysis confirmed that the mold experienced thermal brittleness during use. To enhance mold durability and extend its lifespan, a pulse electrodeposition method was employed to create a NiCo alloy coating as a replacement for the Cr layer on the metal surface. The coating exhibited a smooth and scratch-free surface. The prepared NiCo special coating significantly increased the mold yield strength by approximately 313.8%, facilitated a 13% increase in plastic deformation, and reduced the fracture strain by 25%, effectively preventing mold fracture and improving its service life.

## 1. Introduction

Die casting molds are crucial equipment for the die casting process, and their service life directly affects the cost of finished products. To improve performance and reduce the costs associated with low-pressure castings, higher standards have been set for mold mechanical properties and service life [1,2,3]. Various factors impact the mold service life, including the sound die cast part structure, the innovative design of molds, ideal selection of materials, and advancement and rationality of the manufacturing process. The proper use and soundness of the casting process are necessary, which include selecting the right equipment, alloys, and process parameters. The mold design must consider each stress condition to ensure that the mold meets large-scale production standards [4,5,6]. The tolerance fit and surface roughness of each active part should be adequately selected to avoid metal liquid penetration during the initial operation and to prevent biting under normal working conditions. The design of the filling system, overflow system, and exhaust system should avoid the direct impact or flushing of metal liquid on the mold cavity or core. An effective exhaust guide groove from the overflow slot to the exhaust slot helps to prevent blockage and maintain an efficient exhaust passage [7,8].

To extend the service life of large mold cores, bolt fastening is more preferential than step fastening for easy replacement and convenient operation. The thermal balance of mold parts should be considered in the design, particularly for large and complex molds [9,10,11]. By rationally designing the inlet, overflow, and exhaust systems and by utilizing hot oil type mold temperature control machines, the mold service life can be significantly increased. The casting design should meet the die casting process requirements as much as possible for parts with sharp corners or edges or that are prone to stress concentration. Parts that cannot be avoided should use embedding or splicing to simplify manufacturing, release early cracking and stress concentration, and facilitate maintenance and replacement. Die cast parts should be suitable for the die casting process while meeting the use requirements. Uniform wall thickness and metal liquid flow should not produce heat concentration to accelerate mold thermal fatigue. Fillets should be increased to improve the mold forming part’s stress conditions and avoid sharp edges, corners, and fractures [12,13]. An erosion zone should be left in the pouring port position to extend the mold service life without affecting the casting use requirements. Mold processing technology is constantly evolving but should retain traditional processing technologies and ensure that the surface of the formed part does not have residual machining or scratching marks [14,15,16]. Welding is sometimes necessary but requires attention to material consistency, cleanliness, preheating, insulation, and the adequate removal of welding stress through heat treatment. Reasonable arrangements for heat treatment procedures in the mold manufacturing process directly impact the mold service life [17,18,19,20].

A fracture is a severe defect in the die during use and can significantly impact its service life. Fractures damage the die structure, making it ineffective and unable to function correctly, which can cause the die to fail, necessitating repair or replacement [21,22,23]. This phenomenon can lead to additional costs and disrupt production planning and processes. Fractures can damage the die surface, causing scratches, cracks, and other forms of surface damage, which can impact the appearance of the final product and increase the difficulty of mold maintenance [24,25,26]. Additionally, fractures can modify the material properties of the mold, affecting its hardness, strength, toughness, and other characteristics, ultimately impacting its reliability and service life. For example, defects in the contour design of the mold can negatively affect its fatigue life, while holes or defects in the material can enlarge under stress and lead to premature failure. It is crucial to address fractures promptly by identifying the causes, using effective repair methods, and continuously monitoring and maintaining the mold to extend its service life and ensure high-quality products [27,28,29].

Replacing the original Cr layer with a Ni-Co coating on the mold can improve the wear resistance of the abrasive tool and significantly improve the mold fracture [30,31,32,33]. There have been many studies on the use of pulsed electrodeposition to prepare Ni-Co coatings to improve mold fracture [34,35,36]. Chen Y, Ma H, and Zhang Q et al. [37] investigated the effect of using pulsed electrodeposition to prepare Ni-Co alloy coatings on improving the strength and wear resistance of mold materials. The study showed that the pulsed electrodeposition method can produce high-quality Ni-Co alloy coatings, improve wear resistance, and prevent the fracture of mold materials. Liu Z, Zhang D, and Chen Z et al. [38] investigated the effect of Ni-Co alloy coatings produced via pulsed electrodeposition on improving the properties of mold steel. The experimental results show that the Ni-Co alloy coating can significantly improve the hardness and corrosion resistance of the mold steel, thus effectively improving the fracture problem of the mold. However, there is still little research in the literature on the fracture mechanism analysis of the fracture causes during the production and use of molds, and the targeted use of pulsed electrodeposition on the surface of the original molds to prepare Ni-Co alloy coatings to improve the anti-wear properties of the material. In this paper, the fracture mechanism of small molds fractured in the actual production process was analyzed, and Ni-Co coatings were applied to the surface of the molds using pulsed electrodeposition. The wear resistance of the coated and uncoated molds was then compared. The aim was to improve the wear resistance of the molds and, consequently, their service life.

## 2. Materials and Methods

### 2.1. Detecting the Causes of Mold Fractures in Parts

To detect and analyze the causes of mold fractures in parts, a small broken mold obtained from the actual production process was used as a sample for analysis, and the chemical composition of the mold was measured using an Energy-Dispersive X-ray Fluorescence Spectrometer (EDX-XRF), EDX-LE plus (ID: 61-0069-00002), Shimadzu Co., Ltd. Kyoto, Japan. The purpose of the detection was to conduct a qualitative and semiquantitative analysis of the elemental components in the sample. The detection method involved placing the sample in a sample cup and testing it using the EDX-XRF instrument, and the results were analyzed. The results obtained indicated that the content of each element was within the needed range, which could identify the potential causes of mold fractures. Further analysis and testing could be required to determine the exact causes and provide appropriate solutions to prevent future mold fractures.

The fracture surface was microscopically inspected using SEM, Zeiss Supra 55, Zeiss Co., Ltd., Oberkochen, Germany, and the morphology of the fracture was observed at different magnifications.

### 2.2. Using a Pulse Electrodeposition Method to Prepare NiCo Coatings

To optimize the electroplating qualities of metal surfaces, a new technique using pulse reverse plating to replace the traditional Cr layer with a NiCo layer was implemented. This technique enhanced the electroplating quality and improved the mold durability while preventing problems such as fracture [39,40,41,42]. Here, the mechanism by which the pulse reverse plating technique improved the coating quality was determined:

(1) Uniform coating: The pulse reverse plating ensured highly uniform electroplating deposition, avoiding the formation of thick films and stacking on the workpiece surface.
(2) Dense coating: Positive and reverse pulses were used to deposit and redisperse ions in the plating solution. The pulse reverse plating generated additional reverse pulses, resulting in a uniform distribution of ions and a dense coating to prevent defects.
(3) Reduced coating looseness and holes: The stability provided by the pulse reverse plating reduced the probability of defects, such as loose coatings and holes.
(4) Reduced environmental impact: By reducing the amount of electrolyte needed, the pulse reverse plating minimized the losses of metal ions into sewage and its impact on the environment.

For the experiment, the SMD-500 CNC pulse power supply, Handan Dashun Plating Equipment Co., Ltd., Handan, China, was used. The uncoated mold from the production process was used as the substrate, the same as the mold used in fracture analysis. By referring to relevant works in the literature [43], the chemical materials used in the experiment were determined as follows:

(1) Nickel sulfate heptahydrate NiSO_4_·7H_2_O: The main source of Ni element in the plating layer.
(2) Nickel chloride hexahydrate NiCl_2_·6H_2_O: It has two main roles, which are to provide ions for the plating solution and to increase the conductivity of the plating solution and provide Ni^+^. Cl^−^ in NiCl_2_ can prevent the passivation of the Ni plate and activate the anode.
(3) Cobalt sulfate heptahydrate CoSO_4_∙7H_2_O: The only source of Co^2+^ in NiCo alloys, which can also provide ions for the plating solution and enhance the conductivity.
(4) Boric acid H_3_BO_3_: Boric acid is a weak acid, able to ionize H^+^ in three steps, so adding a certain amount of boric acid in the configuration of the plating solution can make the plating solution pH value maintain stability.
(5) Saccharin C_7_H_5_O_3_NS: Saccharin is a type of brightener that increases the brightness of the plating layer while providing a balance between compressive and tensile stress, resulting in increased ductility. It is also known as the first type of brightener.
(6) Sodium dodecyl sulfate C_12_H_25_SO_4_Na: This is one of the variables in the experiment, and it is used as a wetting agent in the plating solution. The active agent can be adsorbed on the outer layer of the cathode to reduce the surface tension and the possibility of creating pores within the plated layer. If the level is too high, the surface of the plated layer will have an orange peel appearance, which may lead to scrap.
(7) 1,4-Butynediol C_4_H_6_O_2_: This is a secondary brightener used in conjunction with Type I brighteners. It can increase the brightness of the plated layer and, at the same time, increase its toughness.

All chemicals were produced by McLean Biochemical Technology Co., Ltd. Shanghai, China, and were analytically pure. To prepare the electroplating solution, through a literature review and comparative experiments, the optimal ratio and solution preparation process was determined as follows: The following components were added to a beaker containing 50 mL of deionized water: 30.00 g NiSO_4_·7H_2_O, 25.00 g NiCl·6H_2_O, 18.00 g CoSO_4_∙7H_2_O, 20.00 g H_3_BO_3_, 0.25 g C_12_H_25_SO_4_Na, 0.04 g 1,4-butenediol, and 1.50 g C_7_H_5_O_3_NS. The mixture was heated and stirred in a constant-temperature water bath at 60 °C until all the solutes dissolved. Then, 450 mL of deionized water was added, and the electroplating solution was electrolyzed at a current density of 0.5 A/dm^2^ to remove impurities. Finally, the pH was adjusted to 3 by adding a 10% sodium hydroxide solution.

Through a literature review and comparative experiments, the parameters were determined as follows:

(1) Electrodeposition duration: According to the study of the effect of electrodeposition time on the plating properties [44,45], different time variables were set, such as 10 min, 30 min, etc. In this experiment, the electrodeposition time was chosen as 30 min to amplify the effect of time on the plating, and the current was set at 1.5 A.
(2) Duty cycle, Ton/(Ton + Toff): According to the investigation of NiCo alloy deposition rate [46], several gradients of duty cycle were set, including 20%, 30%, 40%, and 60%. Since lower duty cycles are favorable for coating preparation, 20% was chosen as the controls in this experiment, which is Ton at 0.2 ms, Toff at 0.8 ms.

To obtain detailed information about the coating composition, orientation, crystal structure, and surface chemical composition, an AES test was performed. The testing conditions are listed below: instrument signal of PHI 710 AES; electron gun electron beam voltage of 10 kV; and electron beam current of 10 nA. AES testing allows us to determine the elemental composition and surface chemical composition of the coating with high accuracy and sensitivity. By analyzing the energy levels of the emitted electrons, information about the orientation and crystal structure of the coating can be obtained. The results of the AES test can provide additional insights into the structure and composition of the NiCo composite coating that can complement the SEM and EDX analyses.

To further investigate the properties of the NiCo composite coating, a hardness test was performed using a Dynamic Ultramicro Hardness Tester DUH-211S, Shimadzu Co., Ltd., Kyoto, Japan. The test force range was 0.1–200 gf (0.1–1961 mN), and the measuring range was 0.1–10 µm. The microscope magnification was 500×, and the HV range was 100–900. The maximum permissible percentage errors were 4–12%.

The intelligent electronic tensile testing machine, TST-01M, Zhongji electromechanical Equipment Co., Ltd., Jinan, China, was used to test the yield strength (MPa), the plastic deformation (%), and the fracture strain (%) of the coated and uncoated molds.

## 3. Results and Discussion

### 3.1. Analyzing the Causes of Mold Fractures in Parts

Based on the chemical composition measurements in Table 1, it was likely that the type of steel used for the mold was low-carbon steel, as it mainly comprised iron and carbon, with relatively low amounts of other alloying elements, such as chromium, cobalt, and nickel.

**Figure 1:**
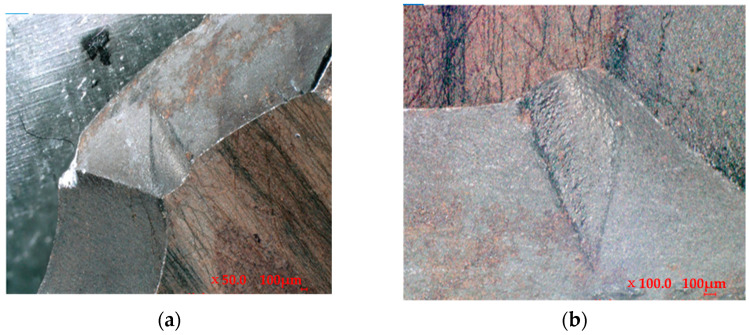
Location of the fracture under a high-power microscope: (**a**) magnified 50 times and (**b**) magnified 100 times.

In addition, from the macroscopic examination of the fracture surface shown in Figure 1, it could be observed that the mold fracture surface was bright and had a distinct metallic luster. The extended ridges with a radial center toward the mold center indicated that the crack source originated at the mold cavity, which rapidly expanded toward the center along the axial direction, resulting in a typical brittle fracture. Therefore, it could be concluded that the mode of mold cracking was a brittle cleavage fracture at the center. Further analysis and testing could be required to determine the exact causes of the mold fracture, including the factors that contributed to the formation of the crack source in the mold cavity and how it rapidly expanded. By identifying the specific causes, appropriate measures could be taken to prevent similar mold fractures in the future and extend the service life of the mold.

**Figure 2:**
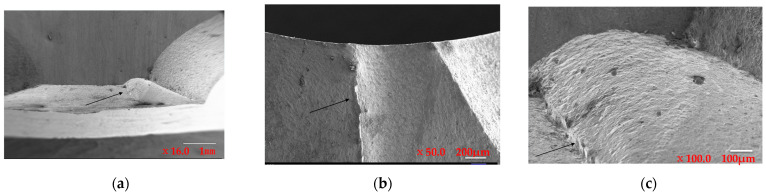

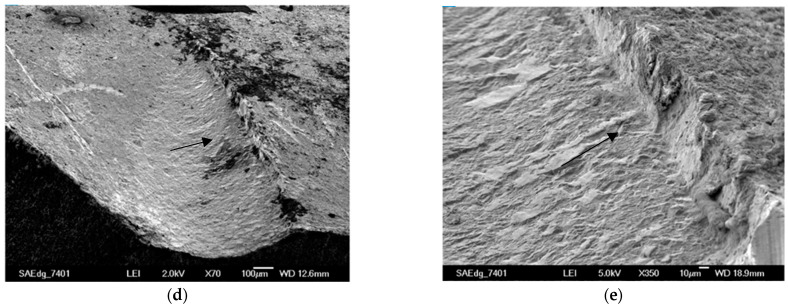
Location of the fracture under SEM at different magnification times: (**a**) 16×, (**b**) 50×, (**c**) 100×, (**d**) 70×, and (**e**) 350×.

As shown in Figure 2. The SEM images of the fracture surface were magnified at 16×, 50×, and 100×, and they clearly show fracture steps, as shown by black arrow, indicating intergranular cleavage fracture characteristics. At high magnifications, such as 100 μm and 10 μm in Figure 2d and Figure 2e, respectively, flat quasi-cleavage planes, micropores, and tear ridges were observed, indicating a fracture along the original grain boundaries.

**Figure 3:**
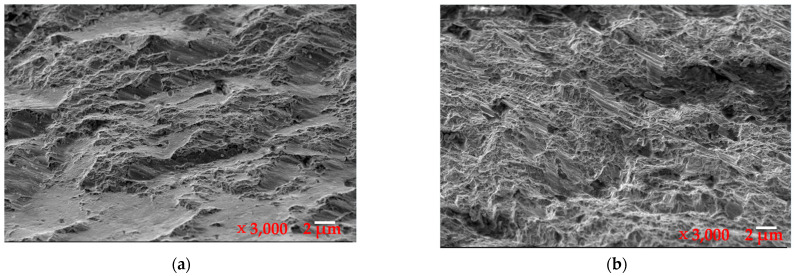
Morphologies of the fracture site and the normal site: (**a**) crack site and (**b**) normal site.

From the high-magnification observations in Figure 3, it was observed that the micromorphology along the grain boundary exhibited ductile dimples. However, the local dimples contained additional nonmetallic inclusions and carbide particles, which had significant concentrations at the microscopic crack sites. This phenomenon corresponded to a quasi-cleavage fracture formed at the concentrations of nonmetallic inclusions and carbides. When comparing the fracture morphology in Figure 3a with the normal site shown in Figure 3b, multiple microcracks were evident, as shown in Figure 3a, which were distributed along the boundaries of the nonmetallic inclusions and carbide particles. This finding indicated that the fractures were caused by the lack of conformity of the nonmetallic inclusions and reticular carbides.

The fracture site in Figure 3a displays microporosity agglomeration and cleavage fracture. Microporosity agglomeration fracture [47,48] is a form of shear fracture, which is a common mode of material toughness fracture. The fracture surface was typically dark gray and fibrous, with numerous dimples distributed on the surface. The process of microporosity agglomeration fracture included micropore nucleation, growth, aggregation, and fracture.

According to the microporosity agglomeration fracture mechanism, the fracture was likely to have occurred at the interfaces between certain phases in the material, such as inclusions and second-phase particles, or at the grain boundaries, twinning regions, phase boundaries, and areas of significant dislocation accumulations under external force. These areas could have formed microcracks. The visible micropores were generated by the agglomeration of adjacent microcracks, and they grew, proliferated, and eventually connected to produce the final fracture.

It is thought that the time of micropore nucleation is determined by the low bonding strength between the second-phase particles and matrix in the material [49,50], and that micropore nucleation typically occurs before necking. Micropore nucleation was considered the primary aspect in controlling the fracture process of martensitic aging steel.

The size and depth values of the dimples on the fracture surface of the martensitic aging steel depend on the quantity and distribution of the second-phase particles and the plastic deformation capability of the matrix. The small and shallow dimples on the fracture surface in Figure 3a suggest that the matrix has a strong work hardening ability [51]. These dimples may be generated due to the specimen loading at the edge, resulting in the σmax not being uniformly distributed across the cross section. The stress at the edge was very high, and the crack gradually increased from the surface to the inside, resembling the process of tearing two stuck papers from one end. Therefore, this phenomenon is known as a tensile tearing type of fracture. Notched or cracked samples often showed this type of fracture surface. The tensile tearing elongated dimple was small and shallow, and the cracks propagated quickly over a macroscopic scale, leading to brittle fracture. This result supported the previous conclusion that the fracture had both brittle and cleavage features.

**Figure 4:**
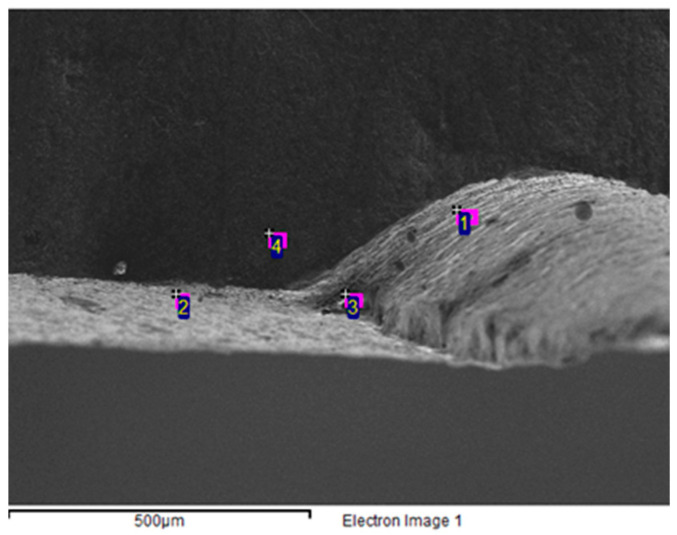
Different positions for the test sample of the crack position.

Four sampling locations, positions 1, 2, 3, and 4 were selected from the fracture surface and analyzed for their composition, as shown in Figure 4. Table 2 summarizes the results of the EDX test carried out on the fracture surface. The EDX test is a technique that enables the identification and quantification of the elemental composition of a sample based on the characteristic X-rays emitted by the sample under the bombardment of high-energy electrons or photons. Analyzing the results of the EDX test is useful for understanding the chemical composition of the fracture surface and identifying any anomalies with respect to the expected composition. This information, combined with other analyses, could provide a comprehensive understanding of the causes of mold fracture and reveal any corrective or preventive measures that are necessary.

The EDX test results presented in Table 2 indicate that oxygen was detected at all four sampling locations on the fracture surface, with the highest oxygen content being 32.6% at location 3. Since the oxygen content in low-carbon steel is typically low, the high oxygen content detected on the fracture surface suggested that the steel surface underwent oxidation or had nonmetallic impurities, such as oxides or manganese oxides, during processing and usage. The mold fabrication, heat treatment, and material selection processes could all contribute to the changes in the oxygen content. However, an overly high oxygen content could decrease the steel toughness and ultimately increase the risks of fracture and oxidation. Therefore, the high oxygen content on the fracture surface could have contributed to the fracture.

Manganese was detected at positions 1 and 2, while no relevant element was detected at positions 3 and 4. This result could suggest that the chemical compositions in these areas were not uniform and could have undergone varying heat treatments. Mn is a common alloying element in low-carbon steel, improving steel hardness and toughness. However, if the concentration of manganese is too high, it could result in an uneven steel strength and increase the susceptibility of these regions to fractures or other damage. These findings could indicate the presence of uneven steel distribution or improper treatment and could have contributed to the mold fracture.

**Figure 5:**
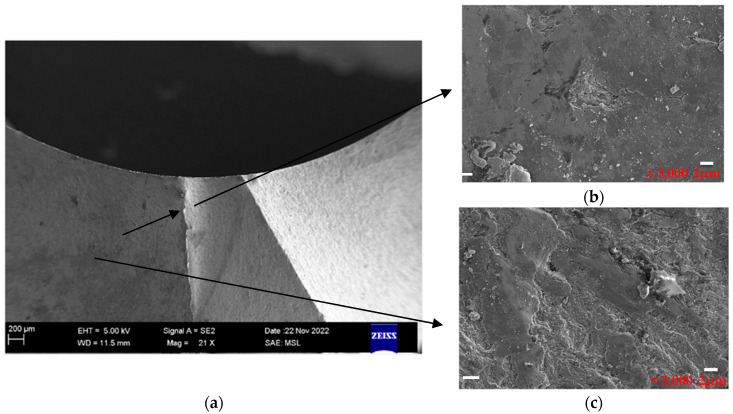

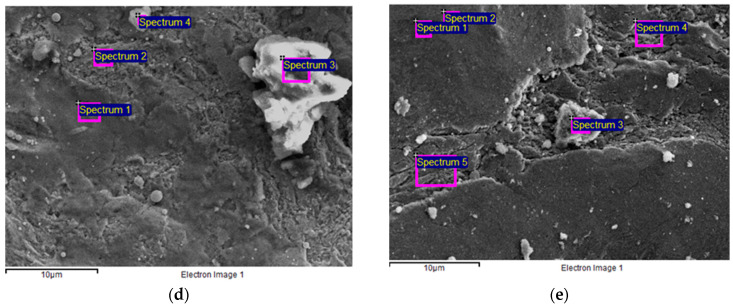
(**a**) SEM images indicating the location of sampling, (**b**) corresponding fracture locations, (**c**) normal locations, (**d**) sampling points of the fracture locations, and (**e**) sampling points of the normal locations.

To compare the composition differences between the fracture and normal surfaces, samples were taken from both specimens for composition testing. Figure 5a shows an SEM image that identified the locations (as shown in black arrow) corresponding to Figure 5b,c. Four locations on the fracture surface were sampled for composition testing, as shown in Figure 5d, while five locations on the normal surface were sampled, as shown in Figure 5e. The EDX comparative test results are presented in Table 3 for the fracture samples and in Table 4 for the normal samples. These results could enable a comparison of the elemental composition of the fracture surface and normal surface and could provide insights into the causes of mold fracture.

A comparison of the composition data obtained from the EDX analysis of the fracture and normal parts of the mold samples revealed that the normal part contained C, O, Si, Fe, Cu, and Zn, while the fracture part contained C, O, Al, Si, P, S, K, Ca, Fe, Co, Cu, Mo, and W. Interestingly, the percentage of Fe in the fracture part significantly decreased. This finding suggested that the mold underwent thermal brittleness during use. Thermal brittleness is a phenomenon causing materials to break easily at high temperatures. This characteristic is often associated with materials absorbing a large amount of oxygen or other impurity elements at high temperatures, leading to changes in the material’s internal structure that reduce its toughness and ductility.

Based on the comparison of the composition data obtained from the EDX analysis of the fracture and normal parts of the mold sample, it was observed that the mold experienced thermal stress during use that resulted in serious fracture phenomena and a significant decrease in the content of elements such as Fe. The type of steel material used for the mold was Cu0.25Si1.21Mn0.86Cr0.07, which is a heavy hardened steel, indicating that the possible presence of Cu led to thermal brittleness. The possible causes of mold fracture could be summarized as follows:

(1) Material internal defects and stress concentration: The presence of different types and sizes of defects, such as inclusions, pores, slags, and internal cracks, could cause stress concentration and expand under stress, leading to brittle fracture. This type of stress concentration could cause the brittle fracture of low-carbon steel molds.
(2) Material quality issues: If the material had uneven quality or inclusions, it could decrease the strength and toughness of the steel, making it increasingly susceptible to fracture under stress. In addition, cracks and corrosion on the surface of the steel could be responsible for static load fracture.
(3) Mold electroplating quality: The quality of the electroplating coating on the mold surface could lead to mold fracture issues. Possible electroplating issues included the following:
  (a) Uneven thickness of the electroplating coating, which could cause an uneven stress distribution on the mold surface, leading to cracks or plastic deformation and affecting the dimensional accuracy and surface quality of the mold;
  (b) A problematic coating structure, which could affect the strength and toughness of the coating, making it prone to cracks and breakage during mold operation;
  (c) A high number of nonmetallic inclusions in the coating, which could affect the density of the coating and cause its toughness to decrease, leading to cracking or breakage;
  (d) The poor quality and surface roughness of the coating, which could attract small impurities, such as air and dust, further affecting its appearance and function; the presence of coating bubbles, delamination, or looseness could reduce the corrosion and protection functions, reducing the service life of the mold.

In summary, to avoid mold fractures, it was important to ensure that the material used for the molds did not have internal defects or stress concentrations and to maintain an even quality and surface finish. Proper quality control was crucial for electroplating coatings, ensuring even thickness, appropriate structure, a low surface roughness, and the absence of nonmetallic inclusions.

### 3.2. Analyzing the Properties of Ni-Co Coating

**Figure 6:**
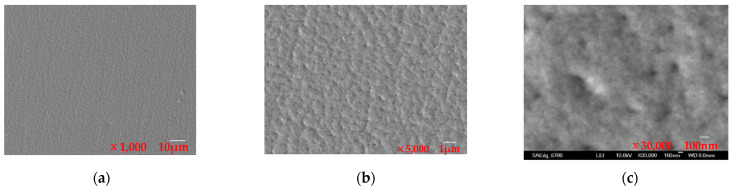
SEM images of the special coating: (**a**) 10 μm, (**b**) 1 μm, and (**c**) 100 nm.

**Figure 7:**
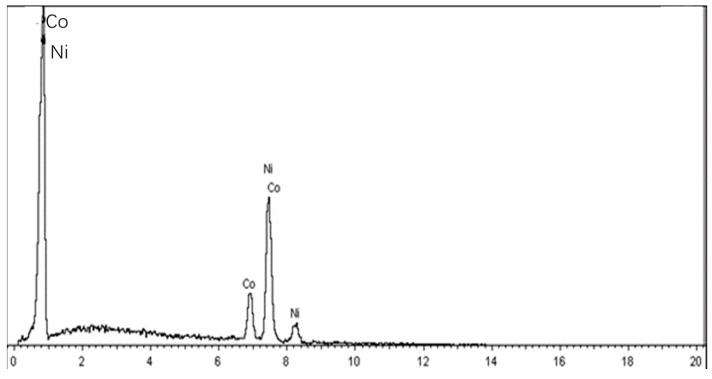
EDX spectrum chart for the special coating.

The SEM images of the special NiCo composite coating are presented in Figure 6, with images at 10 μm, 1 μm, and 100 nm scales shown in Figure 6a–c, respectively. The morphology, roughness, and surface structure of the sample can be observed in Figure 6a at the 10 μm scale, and the surface is found to be very smooth without scratches. Clear details of the coating crystal structure, texture, pores, and spots can be observed in Figure 6b at the 1 μm scale. Details of the morphologies, features, and compositions at the 100 nm scale can be observed in Figure 6c. Furthermore, the EDX spectrum chart for the NiCo coating is shown in Figure 7. The results indicate that the coating comprises approximately 77% Ni and 23% Co.

These results demonstrate that the pulse reverse plating technique used in this experiment leads to the formation of a uniform and dense NiCo composite coating, which is smooth and without defects. The use of this technique enables the control of coating quality and helps to avoid problems such as loose coatings and holes. The EDX results indicate that the composition of the coating is well within the expected range, indicating the effectiveness of the electroplating process.

**Figure 8:**
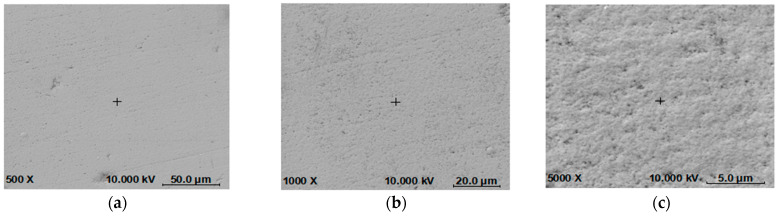

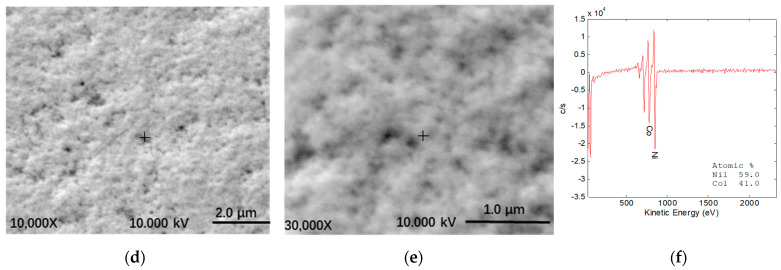
SEM and AES results of the special coating under different magnification ratios. (**a**) 500×, (**b**) 1000×, (**c**) 5000×, (**d**) 10,000×, (**e**) 30,000×, (**f**) AES, sputter 3 min.

The results of the SEM test are shown in Figure 8, with images captured at various magnifications to reveal different features of the coating. At low magnifications, as shown in Figure 8a (500×) and Figure 8b (1000×), the approximate morphology, surface features of the coating, and the roughness of the sample surface can be observed. The coating surface is found to be smooth and flat without scratches. At medium magnifications, as shown in Figure 8c (5000×) and Figure 8d (10,000×), the coating surface can be observed to be smooth with good density, and the details of the coating surface morphology can be observed clearly. In addition, microfeatures such as surface defects and particles can be detected. At high magnifications, as shown in Figure 8d (10,000×) and Figure 8e (30,000×), detailed information about the distributions of the coating composition and features can be observed. The results confirm that the use of the pulse reverse plating technique significantly reduces the looseness and holes in the coating. Particular attention is drawn to Figure 8e, which magnifies the element distribution and interface structure by 10,000 times. The results show that the NiCo composite coating is uniform, and the elemental distribution is highly symmetric. The element mapping indicates that the Ni and Co elements in the coating are evenly distributed, with no significant concentration in any specific area. The analysis suggests that the electroplating process is successful in producing a uniform, dense, and stable NiCo composite coating with good adherence to the metal surface.

The AES test result of Figure 8f provides additional insights into the structure and composition of the NiCo composite coating, and it confirms the effectiveness of the pulse reverse plating technique in achieving the desired electroplating quality.

**Figure 9:**
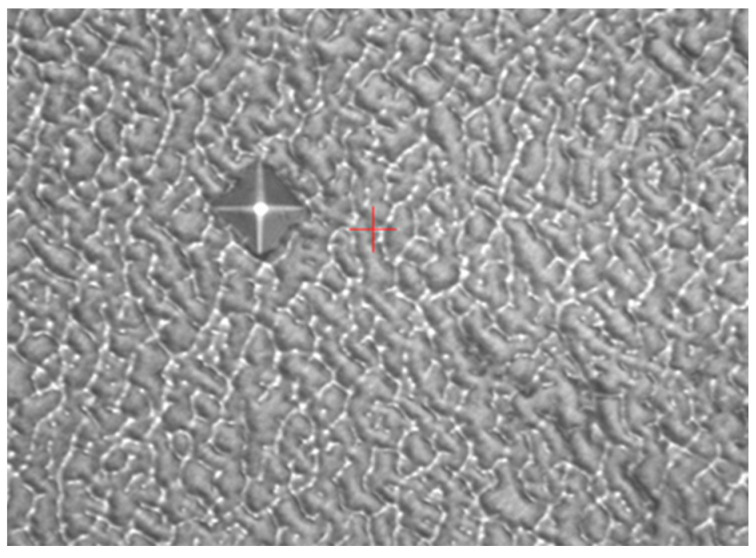
Hardness test image for the special coating.

**Figure 10:**
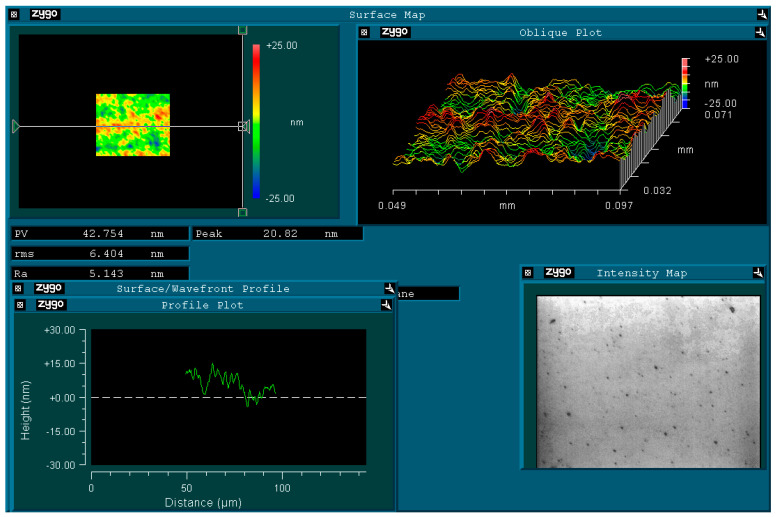
Surface test image for the special coating.

As shown in Figure 9, the hardness obtained is 465.257 HV, which is much harder than the raw materials used for the mold. This result confirms that by using the pulse reverse plating technique, the wear resistance is significantly improved. The application of the NiCo composite coating with improved hardness and wear resistance extends the service life of the mold. This hardness test verifies that the electroplating process using the pulse reverse plating technique leads to the formation of a dense and uniform coating with excellent hardness and wear resistance. The surface roughness of the coating shown in Figure 10 is 5.143 nm, which is relatively smooth with few scratches.

To prevent mold fracture during use, it is important to choose a raw material with high yield strength and low fracture strain. This finding ensures that the mold can withstand a large impact load, and materials with high yield strengths can effectively minimize damage caused by such impact loads, thereby extending the mold service life. However, if the raw material has a fracture strain that is overly high, it may easily fracture, which significantly impacts the mold service life.

Plastic deformation occurs when a material is subjected to a tensile stress that exceeds its yield strength. For mold materials, the degree of plastic deformation should be determined based on the specific usage scenario. In general, mold materials should possess a certain degree of plastic deformation capability to accommodate deformation during use and subsequent recovery. If the plastic deformation ability is overly low, the material may crack and fracture easily, significantly reducing the mold service life. Conversely, if the plastic deformation ability is overly high, the material cannot fully recover from deformation, resulting in permanent damage to the mold.

To enhance the mold service life and work efficiency, the selection of raw material should comprehensively consider the yield strength, plastic deformation, and fracture strain. By striking a balance among these factors, the optimal material for the mold can be chosen. This phenomenon ensures that the mold can withstand impact loads without fracturing, and it possesses the necessary plastic deformation capability to accommodate deformation.

In Table 5, data about several key indicators impacting the mold service life are presented. A comparison of the indicators suggests that the NiCo coating developed in this research significantly increased mould yield strength by approximately 313.8%, moderately increased plastic deformation by approximately 13% and slightly reduced fracture strain by approximately 25%. These findings demonstrate that the NiCo coating can effectively prevent mold fracture and achieve the objective of enhancing the mold service life. For the specific mold that needs to be improved in this experiment, the experimental results are better than those in the literature [46].

## 4. Summary

We presented a comprehensive investigation into the fracture of a mold utilizing advanced techniques, such as energy-dispersive X-ray spectroscopy (EDX), scanning electron microscopy (SEM), and Auger electron spectroscopy (AES). In the analysis, we compared a normal part made of low-carbon steel to a fractured part exhibiting cracks caused by nonconforming nonmetallic inclusions and reticular carbides. The fractures were a result of the agglomeration of microporosity and cleavage fracture. A further examination using SEM and AES was focused on the causes of the mold fracture, particularly emphasizing the significance of dimples on the specimen edge. An EDX analysis confirmed that the mold suffered from thermal brittleness during its operational use.

To enhance the mold durability and extend its lifespan, a pulse electrodeposition method was implemented to create a NiCo alloy coating, replacing the existing chromium (Cr) layer on the metal surface. This specially prepared NiCo coating exhibited a smooth and scratch-free surface. The application of this coating notably increased the mold yield strength by approximately 313.8%, facilitated a 13% increase in plastic deformation, and reduced the fracture strain by 25%. As a result, the mold susceptibility to fracture was effectively prevented, leading to a significant improvement in its overall service life.

In summary, this investigation could provide valuable insights into the fracture mechanisms of molds, offering scientific evidence for the application of pulse electrodeposition and the use of NiCo alloy coatings as effective strategies for enhancing mold durability. These findings have implications for industries relying on molds, contributing to improved manufacturing processes and increased operational efficiency.

## Figures and Tables

**Table 1:** EDX test results.

	Unit	Test Item						
		Fe	Cu	Si	Mn	Cr	Co	Ni
**Sample mold**	(wt.)%	97.609	0.252	1.210	0.855	0.073	NA	NA

**Table 2:** Component testing results at different locations.

wt.%	C	O	Al	Si	S	Cl	K	Ca	Mn	Fe	Co	Cu	Zn	W
1	9.06	10.2		0.9					0.2	79.7				
2	9.46	9.14		0.8					0.2	80.4				
3	7.67	32.6				0.4				57.3	2			
4	14.2	20.2	0	0.6	0.3		0.6	0.3		54.9		6.7	0.9	1

**Table 3:** Components corresponding to different spectrum charts (fracture Sample).

wt.%	C	O	Al	Si	P	S	K	Ca	Fe	Co	Cu	Mo	W
1	9.57	30.1		0.4			0.7	0.4	55.5			1.4	2
2	4.47	2.67		1					91.8				
3	43.9	21.1	0.2	0.2	0.3	0.5	0.3	1.1	8.53		23.9		
4	39.6	16.2	0.2	0.2		0.1	0.2		18.8	0.9	22.7	1.2	

**Table 4:** Components corresponding to different spectrum charts (normal sample).

	C	O	Si	Fe	Cu	Zn
1	4.36	13.19	1.07	81.38		
2	4.23	3.77	1.08	90.92		
3	5.62	14.38	0.72	73.72	4.78	0.78
4	5.36	4.24	1.21	89.18		
5	5.67	3.56	1.11	89.66		

**Table 5:** Comparison of raw materials and mold with different coatings.

	Yield Strength (MPa)	Plastic Deformation (%)	Fracture Strain (%)
Raw materials of mold	420	22	8
Mold with Cr Coating	750	23	8
Mold with Ni-Co coating	1738	25	6

## Data Availability

Data are contained within the article.
